# The Rapid Non-Destructive Detection of Adulteration and Its Degree of Tieguanyin by Fluorescence Hyperspectral Technology

**DOI:** 10.3390/molecules27041196

**Published:** 2022-02-10

**Authors:** Yan Hu, Zhiliang Kang

**Affiliations:** College of Mechanical and Electrical Engineering, Sichuan Agriculture University, Ya’an 625000, China; 2020317020@stu.sicau.edu.cn

**Keywords:** tea, fluorescence hyperspectral technology, adulteration degree, non-destructive

## Abstract

Tieguanyin is one of the top ten most popular teas and the representative of oolong tea in China. In this study, a rapid and non-destructive method is developed to detect adulterated tea and its degree. Benshan is used as the adulterated tea, which is about 0%, 10%, 20%, 30%, 40%, and 50% of the total weight of tea samples, mixed with Tieguanyin. Taking the fluorescence spectra from 475 to 1000 nm, we then established the 2-and 6-class discriminant models. The 2-class discriminant models had the best evaluation index when using SG-CARS-SVM, which can reach a 100.00% overall accuracy, 100.00% specificity, 100% sensitivity, and the least time was 1.2088 s, which can accurately identify pure and adulterated tea; among the 6-class discriminant models (0% (pure Tieguanyin), 10, 20, 30, 40, and 50%), with the increasing difficulty of adulteration, SNV-RF-SVM had the best evaluation index, the highest overall accuracy reached 94.27%, and the least time was 0.00698 s. In general, the results indicated that the two classification methods explored in this study can obtain the best effects. The fluorescence hyperspectral technology has a broad scope and feasibility in the non-destructive detection of adulterated tea and other fields.

## 1. Introduction

Tea is a favorite health drink and one of the most widely used beverages today [1], which contains a range of amino acids, minerals, and vitamins [2]. Research has shown that tea has certain effects on health care and pharmacological impacts [3]. Drinking tea for a long time is good for human health [4]. Tea is becoming more and more popular among consumers worldwide [5].

In recent years, due to globalization and the complexity of the food supply chain, quality and security issues have often arisen, and new and challenging risks have emerged [6]. In the tea market, the problems of tea adulteration, low-quality tea that passes as high-grade tea, and old tea that passes as new tea, also occur from time to time [7]. Tieguanyin contains the highest amino acids, vitamins, minerals, tea polyphenols, and alkaloids [8], which has the most nutritional and pharmacodynamic ingredients compared with other oolong teas, and its price is higher than other oolong teas. Pushed by high economic profits, Tieguanyin adulteration happens occasionally in the market. Some illegal merchants mix Benshan, Maoxie, and Huangjingui in Tieguanyin, their appearance is very similar to Tieguanyin, but has a large price difference in the selling process. It is difficult for ordinary consumers to distinguish the authenticity of tea, which severely infringes consumers’ and operators’ legitimate rights and interests. Under this background, a rapid and non-destructive detection method for the identification of tea adulteration is urgently needed [9].

Currently, many methods, such as gel electrophoresis [10,11], microscopic analysis [12], DNA probe [13], liquid and gas chromatography [14], thermal analysis [15], differential scanning calorimetry (DSC) analysis [16], and composition analysis [17], are often used to detect food adulteration. These methods have clear benefits in the quality inspection of tea, such as high accuracy and quantitative analysis [18]. However, these methods are tedious, laborious, and require various chemical reagents [19], which inhibit the assessment of tea quality in a non-laboratory environment [20]. More importantly, it is very difficult to predict an adulterant mixture ratio. At present, spectroscopy technology has been widely used in food adulteration detection [21]. Near-infrared spectroscopy (NIRS) [22] and Fourier transform infrared spectroscopy (FTIR) [23] have been extensively applied in qualitative analysis, such as authenticity and adulteration [24]. Hyperspectral imaging technology (HSI) has been applied to the classification method [25,26], predicting the composition of agricultural products and detecting food adulteration [21]. To date, the HSI-based non-destructive technology has been widely used in the adulteration detection of honey [27], beef [28], and oil [18]. Although the detection of adulteration has been extensively used in the above research, fewer researchers are paying attention to the detection of tea adulteration. Therefore, it is of great practical value to find a fast and accurate method for the identification of adulteration in tea.

The above spectroscopic techniques greatly improve the efficiency of detection, but they suffer from some problems, such as the peak value they detect is not obvious, too much noise interference, and the experimental detection speed is slow [1]. To address these issues, fluorescence hyperspectral imaging technology (FHSI), which obtains spectral and spatial information simultaneously, can be used. It has the benefits of high selectivity, practical operation, good reproducibility, and practical sampling [29]. Furthermore, the fluorescence hyperspectral system collects images faster than the hyperspectral imaging system. As a novel method of detection, fluorescence hyperspectral technology provides unique advantages for food detection. The basic principle is that when a substance is irradiated by the incident light of a specific wavelength, its molecules absorb light energy and enter the excited state from the ground state, and immediately de-excite and emit emitted light. In this study, there are merely slight differences in the shape, color, and internal components between adulterated tea and pure Tieguanyin, which increase the difficulty of detecting the adulteration of tea. The proportion of adulterated tea samples was in line with the common practice of illegal businessmen in the market. As for the fluorescence characteristics of tea, as a plant, different substances in tea will absorb light in different bands and emit fluorescence in different bands. The previous study [1,30] mentioned the use of fluorescence hyperspectral technology to classify the varieties and grades of tea, which proved the feasibility of fluorescence hyperspectral technology in tea detection. In this study, FHSI is used to detect the degree of tea adulteration to further improve the application of fluorescence hyperspectral technology in tea detection. Consequently, the use of FHSI to detect the degree of adulterated tea is a good choice.

The model proposed int his article is relevant for the quick detection of tea adulteration on the production line, save labor, save test cost, to detect the degree of tea adulteration in the future, to guarantee the authenticity of tea sales, normalize the order of the tea market, and guarantee the legitimate rights and interests of consumers. This study sets up the two-class discriminant model of pure Tieguanyin and adulterated Tieguanyin in the experimental scheme, so as to quickly distinguish adulterated tea from non-adulterated tea; on the other hand, it also sets up the six-class discriminant model that can ensure the rapid identification of tea adulteration under different degrees of tea adulteration. The experimental contents are as follows. Firstly, the fluorescence hyperspectral imaging system obtains spectral information in the 475–1000 nm band. Next, Savitzky–Golay (SG), multiplicative scatter correction (MSC) and standard normal variate (SVN) are chosen to preprocess fluorescence hyperspectral data. Additionally, successive projections algorithm (SPA), competitive adaptive reweighted sampling (CARS), random frog algorithm (RF), and uninformative variable elimination (UVE) are adopted to extract the characteristic wavelengths of the tea sample. Two types of SVM classification models corresponding to the different wavelengths of the information are established for rapid and non-destructive testing.

## 2. Materials and Methods

### 2.1. Selection of Adulterated Tea

**Figure 1:**
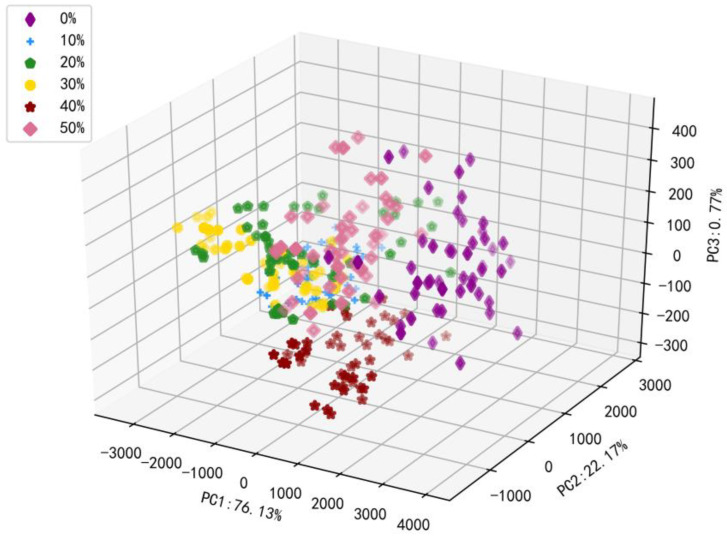
The PCA diagram of pure Tieguanyin and five different adulterated teas.

Tieguanyin (Tie) tea is usually mingled with Huangjingui (Huang), Benshan (Ben), and Maoxie (Mao) in real sales. The choice of adulterated tea is of vital importance, and there are two reasons for this. Firstly, according to the appearance, all teas chosen for adulteration are of dense particles. Huang tea is yellow and the fragrance is sweet-scented osmanthus; it is easy to distinguish and, therefore, it is rarely used as an adulterated tea. Some of Mao teas have the appearance of hairy clusters on dry tea, but not all of them appear, so some businessmen choose it as an adulterated tea. Ben tea is the “close relative” to Tie tea, known as Tie’s brother, and is one of the four well-known oolong teas in China. Moreover, due to its strong growth adaptability, Ben tea’s trading value is less than Tie’s. Secondly, according to figures of PCA and t-SNE when reduced to 2 and 3 dimensions, it can be seen that there were lots of intersections between Tie and Ben, as is described by Hu et al. [1]. In summary, using Ben as an adulterated tea was useful to this study. Figure 1 illustrates the PCA diagram of pure Tieguanyin and five different adulterated teas. Corresponding to the first three PCs of the data set, PC1 accounts for 76.13%, PC2 accounts for 22.17%, PC3 accounts for 0.77%, and the cumulative information variance contribution reaches over 99.07%. On the one hand, the diagram contains most of the information of the original data; on the other hand, the distribution diagram of pure Tieguanyin and adulterated tea can be seen from the diagram. The analysis provided a foundation for the following classification models.

Tie and Ben samples were obtained from Anxi County, Quanzhou City, Fujian Province, China. The types of tea were provided by the merchants. In order to ensure the accuracy of tea samples, these tea samples were sent to the Ya’an tea experts for identification in advance. After that, the verified tea was sent to the laboratory for fluorescence hyperspectral image acquisition.

According to the survey, the proportion of adulteration was generally less than 50% in actual sales. There are many cases of adulteration of tea in the market, but in fact, directly purchased tea cannot be used for accurate adulteration detection experiments. Therefore, six proportions of Tie (100%, 90%, 80%, 70%, 60%, and 50% of total weight) were mixed with six different proportions of Ben (0%, 10%, 20%, 30%, 40%, and 50% of total weight), with each sample weighing 5 g; each sample was measured by a high-precision electronic scale. The proportion of adulterated tea is shown in Table 1. Each proportion of tea consists of 48 samples. In all, 288 samples were collected. 

### 2.2. Fluorescence Hyperspectral Image Acquisition

As a novel method of detection, fluorescence hyperspectral technology provides unique advantages for food detection. The basic principle is that, when a substance is irradiated by incident light of a specific wavelength, its molecules absorb light energy and enter the excited state from the ground state, and immediately de-excite and emit emitted light. In this study, after absorbing the appropriate light energy, the covalent electrons of the tea to be tested in the ground state transition from the bonded molecular orbital or non-bonded molecular orbital pass to the anti-bonded molecular orbital to form the molecular excited state. The excited state of the molecule is volatile and can soon disintegrate. When a molecule returns to its excited state, it is often accompanied by photonic radiation. This is the mechanism for emitting fluorescence from tea.

In this study, the fluorescence hyperspectral image of tea was captured by the GaiaFluo(/Pro)-VN-HR series of fluorescence hyperspectral testing system produced by Sichuan Dualix Spectral Image Technology Co., Ltd. (Sichuan, China). The hyperspectral camera provides the benefits of a high signal-to-noise ratio and sensitivity in the band of 350–1100 nm. The spectral resolution is 2.8 nm, and each hyperspectral pixel is 2048 × 946. Within the system, the excitation light source provided an incident light source to excite the sample, the xenon lamp light source was used as the excitation light source of the fluorescence imaging system, and the detectable spectral range was 350–1000 nm. Before the acquisition of the fluorescence hyperspectral image, a fluorescence filter was used to eliminate the main error of fluorescence measurement from stray light and scattered light; through the multiple combination of excitation filter and fluorescence filter, it is found that under the irradiation of four different wavebands of excitation light sources in the laboratory, the 390 nm excitation filter could be better cut off the light input of other bands. Under the effect of the excitation light source, particular attention must be paid to the fluorescence signal of the sample. Therefore, selecting the 495 nm fluorescence filter can complete the separation of the fluorescence signal and other light, so that the sample captured by the hyperspectral camera can produce the best fluorescence signal.

**Figure 2:**
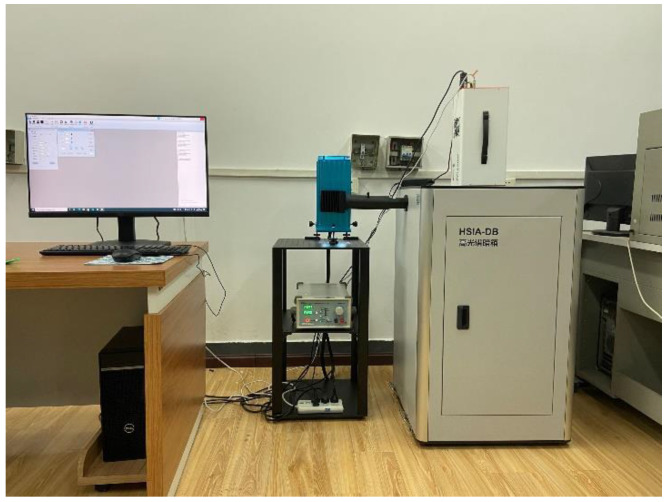
The fluorescence hyperspectral system.

The experiment was performed at 27 °C room temperature and 50% room humidity. The RGB channels of the collected fluorescent images were 638, 551, and 442, respectively, the moving speed of the system was 0.26 mm/s, and the camera exposure time was 0.8 s. The fluorescence hyperspectral system is shown in Figure 2. The tea samples were placed in a 30 × 20 × 30 mm^3^ rectangular transparent container containing 5 g of each sample. After mixing evenly, they were spread on the bottom firmly and evenly.

### 2.3. Region of Interest Extraction

Before analyzing the fluorescence spectra, it is very important to make a reasonable choice of the region of interest, which is directly related to the quality of the extracted data. In this experiment, the spectral data of ROI were extracted through ENVI 5.3 [1]. A rectangular region of interest around the tea sample was selected, and the mean value of the region value was taken as the sample spectral data. Thus, the extracted data covered the whole tea sample and avoided the edge of the sample disk, which can effectively reflect the information from the entire tea sample [31].

### 2.4. Spectral Pretreatment

In the process of collecting fluorescence hyperspectral data [32], the experimental environment, instrument influence, and individual differences of test samples have some interference with the collected data [33]. The interference causes a lot of noise in the data that inevitably affects the establishment and speed of the model. Hence, before modeling, it is necessary to reduce noise interference on the original data [9]. Three types of preprocessing methods were used in this study. Multivariate scattering correction (MSC) [34] and standard normal variable (SNV) [35] made it possible to effectively reduce the impact of baseline drift, tilt, and other noises. Savitzky–Golay smoothing (SG) [2] could efficiently reduce noise, correct spectral baseline, reduce background interference, and enhance spectral resolution. In this study, the 7-point SG smoothing was used [36]. After a comparison of the different methods of preprocessing, the three above often give better results than the other methods. The preprocessing methods were compared through the evaluation indexes of the SVM classification model.

### 2.5. Spectral Characteristic Wavelength Selection

Based on the above preprocessing method, it was considered that the fluorescence hyperspectral data not only contained abundant sample information, but also a larger amount of data and higher data dimensions. Thus, if we directly used high-dimensional data, its huge amount of data dimensions would have led to a surge in computing and an increase in computing time, which would hinder the application of the model in practical production. In this study, the dimension of the data could be reduced by the selection of the characteristic wavelength, so SPA, CARS, RF, and UVE were used to solve this problem.

Successive projections algorithm (SPA) is a forward selection algorithm [34]. It is a useful tool for selecting small variable quantum sets with less collinearity [37]. In general, a wavelength signal is randomly selected as the initial criterion, and then the iteration begins. Each iteration will merge a new band until the specified number of wavelengths is reached, and then the iteration ends. Each iteration selects the band with the least collinearity with the currently selected band; the collinearity between wavelengths may be eliminated to reduce the redundancy between wavelengths and improve the signal-to-noise ratio.

Competitive adaptive reweighted sampling (CARS) is a simple and effective variable selection method [38]. It adheres to the strategy of “survival of the fittest” to select feature bands. Bands with the highest absolute value of regression coefficient were selected from the PLS model using a rebalanced adaptive sampling technique, iteratively and competitively. N subsets of variables are disposed of using N samples, and the subset with the lowest RMSECV value is known as optimal feature bands [36].

Random frog algorithm [38] is an iterative method of variable selection for randomized selection [39]. First, a subset of variables is selected. For each iteration, the selected subset is updated. After N iterations, the probability of selecting a variable is calculated. Finally, PLS is established for variables with high probability, and the variable with low RMSECV in the model is selected. As a variable after the screening, this method demonstrates greater efficiency in the selection of wavelengths and other algorithms.

Uninformative variable elimination (UVE) is a variable selection algorithm to eliminate noninformative variables and perform feature extraction [40]. Its basic principle is based on the regression coefficient of the partial least squares algorithm, taking the ratio of the mean and standard deviation of the regression coefficient of each variable as the evaluation index of variable importance (effective information); a random noise matrix of the same type as the data set is then constructed, the important index of the noise matrix is calculated, which is used as the threshold to judge the effective information, and the remaining variables are selected as the characteristic variables.

### 2.6. Support Vector Machine (SVM)

SVM [41] is a classification model that splits samples into hyperplanes [42]. The segmentation principle is the interval between samples from different classes. For nonlinear problems, the training samples can be mapped from the original space to the high-dimension space, so that the samples can be linearly separable into the space. The kernel function can significantly simplify the computational complexity of high-dimensional problems. After selection, the ‘poly’ kernel function has high accuracy, so all classification models choose it as the kernel function.

### 2.7. Performance Evaluation of the Model

In the 2-class discriminant models, the statistical classification accuracy of adulterated tea was obtained by measuring overall accuracy, sensitivity [43], and specificity. Sensitivity indicated the discrimination on pure Tie, and specificity indicated the discrimination on the adulterated tea.

(1) $$\mathrm{Overall}\;\mathrm{accuracy}=\frac{\mathrm{TP}+\mathrm{TN}}{\mathrm{TP}+\mathrm{TN}+\mathrm{FP}+\mathrm{FN}}$$

(2) $$\mathrm{Sensitivity}=\frac{\mathrm{TP}}{\mathrm{TP}+\mathrm{FN}}$$

(3) $$\mathrm{Specificity}=\frac{\mathrm{TN}}{\mathrm{TN}+\mathrm{FP}}$$

TP is the number of true positives (pure Tie), FN is the number of false negatives (adulterated tea), and TN is the number of true negatives (adulterated tea). FP is the number of false positives (pure Tie). TP and TN represent correctly classified tea; FP and FN represent wrongly classified tea; TP and FN correspond to pure Tie samples. Conversely, TN and FP correspond to the adulterated samples.

In the 6-class discriminant models, the evaluation indexes of adulterated tea are obtained by measuring class accuracy and overall accuracy [44].

(4) $$\mathrm{Class}\;\mathrm{accuracy}=\frac{“\mathrm{Number}\;\mathrm{of}\;\mathrm{correct}\;\mathrm{assignment}\;\mathrm{of}\;\mathrm{each}\;\mathrm{class}”}{“\mathrm{Total}\;\mathrm{sample}\;\mathrm{number}\;\mathrm{of}\;\mathrm{each}\;\mathrm{class}\;\mathrm{tested}”}$$

Time is an indicator to measure the rapidity of the two classification models. The time is consumed for the whole model.

The feature selection method was completed on MATLAB 2018a software platform; other programs were completed on the Python 3.6 platform; and Origin 2017 software was used applied for drawing.

## 3. Results

### 3.1. Characterization of Fluorescence Spectra

**Figure 3:**
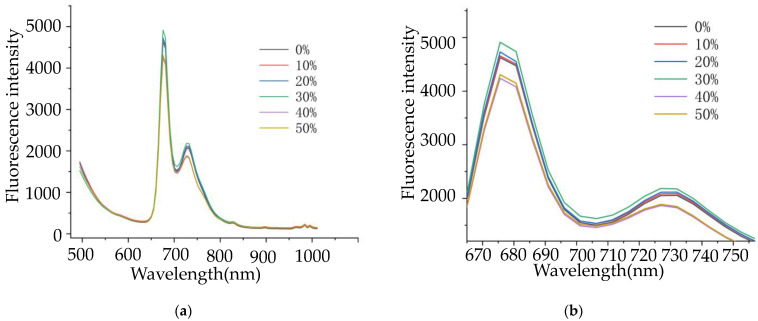
Spectra of tea samples. (**a**) The mean of initial spectra of tea; (**b**) a partially magnified view of (**a**).

Spectral data were collected by a fluorescence hyperspectral system, and a total of 288 tea samples were obtained. The samples were similar in the whole spectral range of 475–1000 nm, and there was no difference in the spectral trend after adulteration [45]. Tea has the fluorescence characteristic of plants, chlorophyll is a key pigment in tea photosynthesis, and the fluorescent signal may reflect the information of chlorophyll [46]. Figure 3 illustrates the mean spectral values of tea samples. There are two evident peaks, about 690 nm and 735 nm, which happened because chlorophyll has a bimodal distribution in the near-infrared region. Different chlorophyll levels lead to different fluorescence intensities; the fluorescence intensity of 500–650 nm is not high, probably due to the synthetic reflection of catechins, theaflavins, and anthocyanins. The low fluorescence intensity of nearly 680 nm is attributed to the high absorption of chlorophyll by plants in the red-light range of 650–690 nm, which is characteristic of green plants. The reason for the fluorescence intensity trend in tea is similar to those reported by Y. Li et al. [30]. The differences in the content and internal structure of Tie and Ben tea lead to different fluorescence intensities in the fluorescence spectra of the two types of tea. It was difficult to distinguish pure and adulterated tea with the naked eye, which provided a basis for identifying the adulteration.

### 3.2. Result of Spectral Preprocessing

**Figure 4:**
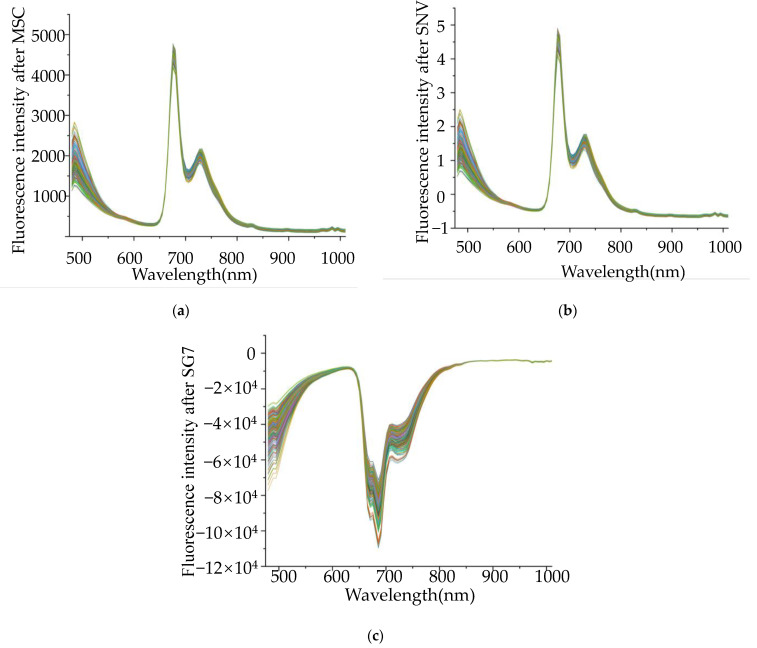
Three preprocessing methods: (**a**) MSC; (**b**) SNV; and (**c**) SG7.

Figure 4a–c shows that all samples are preprocessed by SNV, MSC, and seven-point SG smoothing. Compared to the original spectra, the spectral data processed by SNV and MSC were the same as the original spectral trends, but the noise interference was reduced. This is because the algorithm reduces diffusion interferences caused by different particle sizes. In this study, due to there being no theoretical research for the selection of smoothing times, seven-point SG smoothing was used for preprocessing, which had the well-done effect reported by Tao et al. [7]. After SG smoothing, the data in Figure 4c seem smoother, and the data noise from the data was also reduced.

### 3.3. Result of Classification

#### 3.3.1. Two-Class Discriminant Models

As mentioned above, four different preprocessing methods combined with SVM were established to distinguish pure Tie and adulterated tea. The two- and six-class discriminant models were similar to those of the detection of the cassava flour adulterants reported by Tao et al. [7]. A total of 288 samples were used in each model, including 48 pure Tie and 240 adulterated tea samples. Before the classification model was established, the samples were divided into calibration and prediction according to 2:1. Finally, the performance of the model was assessed on overall accuracy, sensitivity, and specificity.

From the distribution of tea in Figure 1, pure Tieguanyin and adulterated tea can be distinguished, but further analysis is needed to further classify these two types of tea. The results of the establishment of the classification model are presented in Table 2. SNV and MSC had similarities in processing methods, which were: they eliminated the scattering effects caused by uneven particle distribution and different particle sizes so that the accuracy obtained in the classification model was also similar. Compared to seven-point SG smoothing, sensitivity, specificity, and accuracy were improved. Thus, in the four preprocessing methods, the effect of the seven-point SG smoothing had a good effect in distinguishing pure Tie and adulterated tea. In terms of the time consumed, regardless of the preprocessing used, the consumption time was about 2 s. The accuracy of various preprocessing models to distinguish pure Tie and adulterated tea was between 95~100%. The specificity lied between 98% and 100%, and the sensitivity was between 75% and 100%. However, the models using different types of preprocessing were different. When the data were input into the classification model without preprocessing, the specificity of the data reached 100%, but the sensitivity was the lowest. It did not accurately predict the number of pure Ties, which may not meet the ideal requirements. When the preprocessing method was added, the sensitivity had been greatly improved. It can be seen that all the evaluation indexes of SNV and MSC in the calibration had reached 100%, and that of the prediction was above 90%. Among all preprocessing methods, the seven-point SG smoothing had the best effect; it took less time and its sensitivity, specificity, and accuracy reached 100%. Therefore, the seven-point SG smoothing was used in the following two-class discriminant models.

**Figure 5:**
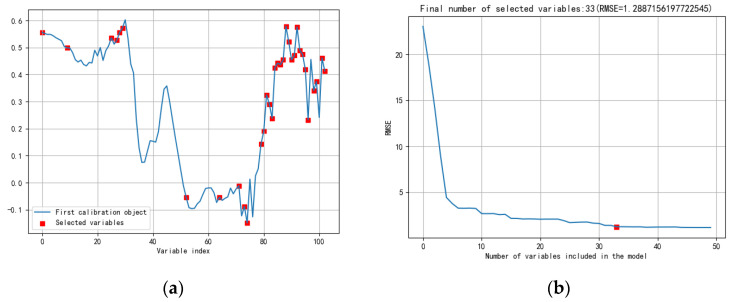
Feature selection after SG-SPA. (**a**) Selected variables (**b**) Number of variables included in the model.

**Figure 6:**
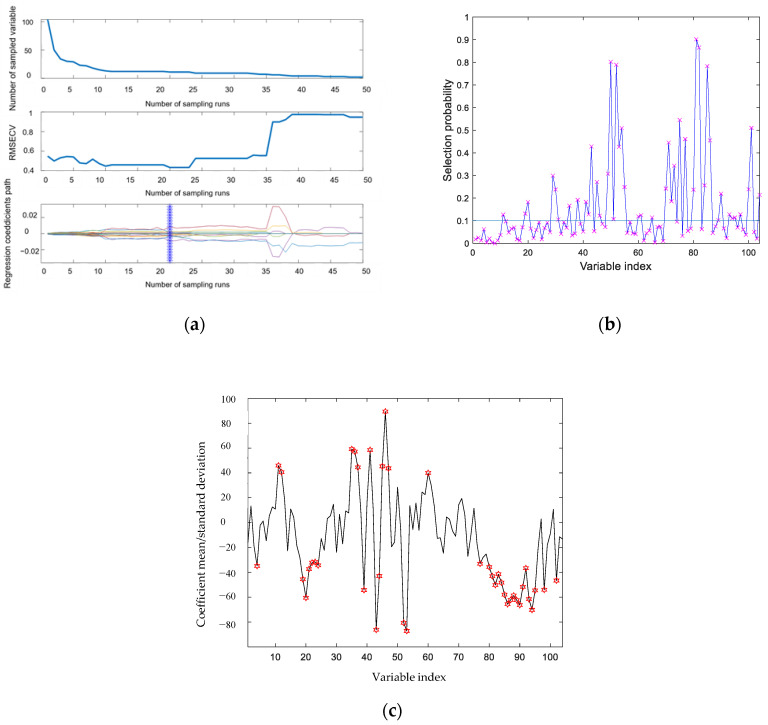
(**a**) Feature selection after SG-CARS; (**b**) feature selection after SG-RF; and (**c**) feature selection after SG-UVE. (The “×” and the star in figure are the selected variables).

After the determination of the preprocessing method of the model, the characteristic wavelength was selected to simplify the model. After SG smoothing, four feature selection methods were applied. Seven-point SG smoothing combined with SPA, CARS, RF, and UVE algorithms reduced the 104 channels to 33, 11, 44, and 46 channels, respectively. Figure 5 shows the feature selection after SG-SPA. Figure 6a shows the feature selection after SG-CARS; Figure 6b,c show the feature selection after SG-RF, SG-UVE. The evaluation indexes of the seven-point SG smoothing combined with four characteristic wavelength selection methods are shown in Table 3. All of the feature selection methods were helpful to reduce the data dimension. Both SPA and RF contain about 40 wavelength bands, and the evaluation indexes detected in the prediction were worse than those obtained without the feature selection method, which was not conducive to the establishment of the model. However, when using UVE, all its evaluation indexes reached 100%, which was the same as the results after CARS, but its features were far more than the features selected by CARS. From the selected bands, it can be seen that CARS had the least number of bands, but it had the greatest accuracy among these characteristic band methods. In total, 11 bands were selected, indicating that CARS has more benefits, including fewer features but more informative bands. The bands of 650, 727, 737, 742, 748, 867, 889, 894, 910, 915, and 995 nm were selected. The bands were concentrated within 650–995 nm, which fully reflected the benefits of the algorithm’s band selection. In terms of all evaluation indexes, such as the feature numbers, and time used, the effect of SG-CARS-SVM was the best, taking only 1.2088 s, with an accuracy of 100%, sensitivity of 100%, and specificity of 100%. Our result is superior to those in the study reported by Tao et al. [7], in which the best overall accuracy of the two-class discriminant model was 97.53%.

#### 3.3.2. Six-Class Discriminant Models

In Figure 1, although pure Tieguanyin and adulterated tea may be distinguished to a certain extent, it was still difficult to classify different grades of adulterated tea.

Firstly, as in the two-class discriminant model, four types of models were established by combining different preprocessing methods with SVM. Then, to simplify the model, four feature selection methods were selected to screen the feature wavelength of the model, which was conducive to improving the effect and accuracy of the model. Results for all models are shown in Table 4. Among these, there were differences between the preprocessing method of the six-class discriminant model and the two-class discriminant model. The accuracy of the classification model was enhanced through the use of SNV and MSC. Both of them had an accuracy of 93.18% without feature selection, which had an advantage by following the feature selection of the model.

**Figure 7:**
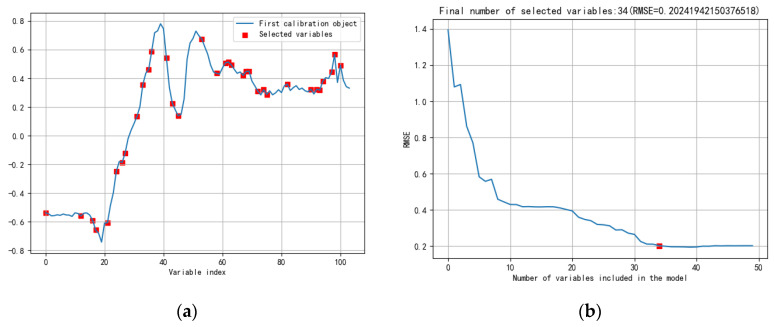
Feature selection after SG-SPA. (**a**)Selected variables (**b**) Number of variables included in the model.

**Figure 8:**
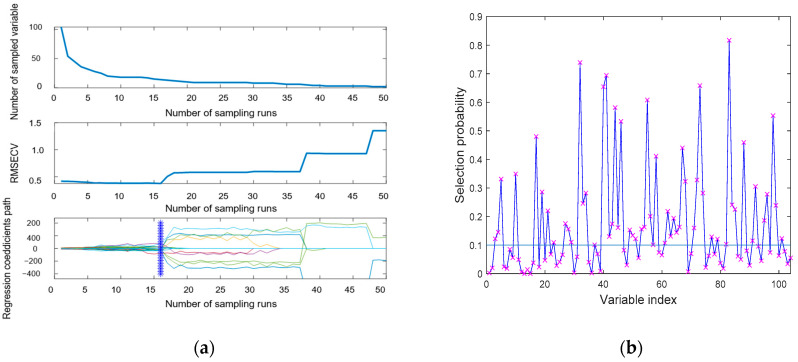

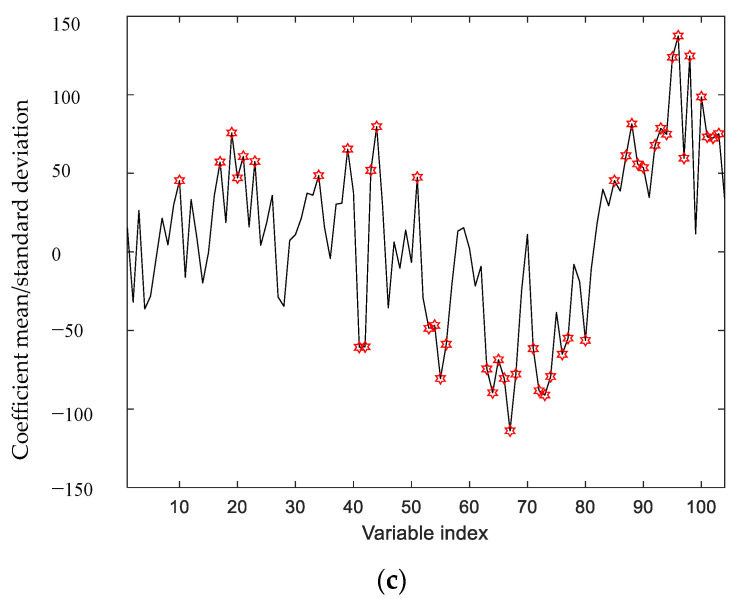
(**a**) Feature selection after SNV-CARS; (**b**) feature selection after SNV-RF; and (**c**) feature selection after UVE-SNV. (The “×” and the star in figure are the selected variables).

The general trend of these models after feature selection was roughly the same. The accuracy of pure Tie, and 10% and 30% of adulterated tea was almost 100%, but the accuracy of 40% was not high, indicating that the model may not accurately distinguish this adulteration level of Tie. In contrast, following the SNV, the overall accuracy of the model was enhanced. Figure 7 shows the feature selection after SNV-SPA. Figure 8a shows the feature selection after SNV-CARS. Figure 8b,c show the feature selection after SNV-RF and SNV-UVE. When the feature selection method was added, all models ran less time, and the benefits of feature selection for the model were more fully realized. After CARS, the number of features were collected and the running time of the model were the least, indicating that the reduction in the number of features is conducive to the realization of the index of rapidity. Following the RF, the overall accuracy was improved by 1.09% and the time difference was only 0.002 s. In this classification, pure Tie and 10% and 30% of adulterated tea were accurately predicted; the accuracy of the remaining proportion of adulterated tea was also improved with the change in the model. Considering the overall effect, RF demonstrated greater efficiency in establishing the classification model. In all, the best method of distinguishing pure Tie and adulterated tea was SNV-RF-SVM, whose overall accuracy was 94.27% and took only 0.00698 s.

## 4. Conclusions

Fluorescence hyperspectral imaging technology combined with SVM can identify 0%, 10%, 20%, 30%, 40%, and 50% of adulterated tea and pure Tie. In the two-class discriminant model, the best overall accuracy was 100%, the best specificity was 100%, the best sensitivity was 100% and the time was 1.2088 s. In the six-class discriminant model, the potential of the model established in detecting doping levels can be found from the perspective of the several designed doping level gradients. The detection accuracy of tea with an adulteration rate of 40% was not very good, and the best accuracy of SNV-RF-SVM was only 72.73%. Six kinds of adulterated tea mixtures were analyzed, a total of 96 tea samples were analyzed; the measurement accuracy was 94.27%, which took only 0.00698 s. From the comparison between the two- and six-class discriminant models, on the whole, the effect of the two-class discriminant models was significantly better than that of six, which showed that the established model can accurately identify pure Tie and adulterated tea. Then, because of the addition of the adulteration proportion, the difficulty of model recognition increases. However, the overall effect was satisfactory, which showed that it is possible to use fluorescence hyperspectral technology as a rapid and non-destructive method to detect adulterated tea. Compared with the research reported by Tao et al. [7], the experimental method of this study added time parameters to evaluate the rapidity of the model. In addition, it can achieve 100% accuracy in the two-classification model and better improve the efficiency of the model.

With the improvement of living standards, people pay more and more attention to food safety. While studying the application of this technology in tea, it also provides a new idea for food detection. On the one hand, as a new technology, FHSI has the advantages of being non-destructive, highly efficient, highly sensitive, and monitored in real time. In this study, two adulteration detection schemes were designed: the two-class discriminant is the classification of pure Tieguanyin and adulterated tea, and the six-class discriminant model can identify the adulteration degree of tea leaves and provide new ideas for the design of adulteration schemes in the future. On the other hand, fluorescence hyperspectral imaging technology can be combined with a variety of algorithms. Combined with this research scheme, preprocessing and feature selection methods can reduce noise interference, extract effective bands and improve the operation efficiency of the model in spectral processing, which further proves the superiority of the research scheme. In future research, we will focus on the deep detection of adulteration by mixing other similar tea leaves into Tieguanyin tea. This will also provide research ideas for the detection of other kinds of tea adulteration.

## Figures and Tables

**Table 1:** The proportion of adulterated tea.

Label	Tieguanyin	Benshan
0	100%	0%
1	90%	10%
2	80%	20%
3	70%	30%
4	60%	40%
5	50%	50%

**Table 2:** The evaluation indexes of different preprocessing methods.

Methods		Sensitivity	Specificity	Accuracy	Time
**RAW**	Calibration	75.86%	100.00%	95.63%	1.9588
	Prediction	84.21%	100.00%	96.25%	
**SNV**	Calibration	100.00%	100.00%	100.00%	2.1267
	Prediction	89.47%	100.00%	97.50%	
**MSC**	Calibration	100.00%	100.00%	100.00%	1.7759
	Prediction	94.74%	98.36%	97.50%	
**SG-7**	Calibration	100.00%	100.00%	100.00%	1.7861
	Prediction	100.00%	100.00%	100.00%	

**Table 3:** The evaluation indexes of the seven-point SG smoothing combined with four feature selection methods.

SG7	Number		Sensitivity	Specificity	Accuracy	Time (s)
**SPA**	41	Calibration	100.00%	100.00%	100.00%	1.2147
		Prediction	98.51%	100.00%	98.75%	
**CARS**	11	Calibration	100.00%	100.00%	100.00%	1.2088
		Prediction	100.00%	100.00%	100.00%	
**RF**	44	Calibration	100.00%	100.00%	100.00%	1.1935
		Prediction	100.00%	94.74%	100.00%	
**UVE**	41	Calibration	100.00%	100.00%	100.00%	1.1829
		Prediction	100.00%	100.00%	100.00%	

**Table 4:** Results for all models.

Preprocessing	Methods	Number	Class Accuracy						Overall Accuracy	
			0%	10%	20%	30%	40%	50%		Time
**RAW**	NO	104	100.00%	100.00%	100.00%	100.00%	59.09%	100.00%	93.18%	0.01396
	SPA	33	100.00%	100.00%	78.57%	81.82%	45.45%	100.00%	84.31%	0.01396
	CARS	19	100.00%	100.00%	78.57%	100.00%	36.36%	78.57%	82.25%	0.01296
	RF	60	100.00%	100.00%	78.57%	100.00%	36.36%	78.57%	82.25%	0.01396
	UVE	41	100.00%	100.00%	78.57%	100.00%	45.45%	100.00%	87.34%	0.01300
**MSC**	NO	104	100.00%	100.00%	92.86%	100.00%	68.18%	100.00%	93.51%	0.01097
	SPA	34	100.00%	94.74%	64.29%	100.00%	54.55%	78.57%	82.03%	0.00801
	CARS	11	100.00%	100.00%	71.43%	72.73%	40.91%	78.57%	77.27%	0.00798
	RF	55	100.00%	100.00%	71.43%	100.00%	63.64%	92.86%	87.99%	0.00898
	UVE	34	100.00%	100.00%	71.43%	100.00%	59.09%	85.71%	86.04%	0.00997
**SNV**	NO	104	100.00%	100.00%	92.86%	100.00%	68.18%	100.00%	93.51%	0.00798
	SPA	27	100.00%	100.00%	85.71%	81.82%	54.55%	85.71%	84.63%	0.00698
	CARS	14	100.00%	100.00%	78.57%	100.00%	45.45%	71.43%	82.58%	0.00499
	**RF**	**57**	**100.00%**	**100.00%**	**92.86%**	**100.00%**	**72.73%**	**100.00%**	**94.27%**	**0.00698**
	UVE	46	100.00%	100.00%	64.29%	100.00%	59.09%	85.71%	84.85%	0.00698
**SG**	NO	104	100.00%	100.00%	85.71%	100.00%	45.45%	100.00%	88.53%	0.00898
	SPA	41	100.00%	100.00%	71.42%	72.73%	31.82%	71.43%	74.57%	0.00798
	CARS	11	100.00%	100.00%	92.86%	72.73%	45.45%	100.00%	85.17%	0.00698
	RF	44	100.00%	100.00%	78.57%	90.91%	45.45%	92.86%	84.63%	0.00798
	UVE	41	100.00%	100.00%	78.57%	81.82%	36.36%	85.71%	80.41%	0.00898

## Data Availability

The data can be found here: https://github.com/guyueguyue/guyuea/blob/main/data2 (accessed on 10 January 2021).

## Sample Availability

Samples of the compounds are not available from the authors.
